# Assessment of the Effectiveness and Predictability of Maxillary Molar Distalization with Clear Aligner Systems: A Systematic Review of the Literature

**DOI:** 10.3390/jcm15145568

**Published:** 2026-07-15

**Authors:** Pau Badía i Martí, Carlos Bellot-Arcís, Beatriz Tarazona-Álvarez, Vanessa Paredes-Gallardo, Natalia Zamora-Martínez

**Affiliations:** Department of Orthodontics, Faculty of Medicine and Dentistry, Universidad de Valencia, c/ Gascó Oliag, 1, 46010 Valencia, Spain; pau.bamar01@gmail.com (P.B.i.M.); carlos.bellot@uv.es (C.B.-A.); beatriz.tarazona@uv.es (B.T.-Á.); vanessa.paredes@uv.es (V.P.-G.)

**Keywords:** clear aligners, molar distalization, effectiveness, predictability, attachments, auxiliaries

## Abstract

**Background/Objectives**: Maxillary molar distalization is a commonly used orthodontic approach for correcting dentoalveolar Class II malocclusions. However, the predictability of such movements when performed with clear aligners remains uncertain. This systematic review aimed to identify the effectiveness and most predictable protocol for achieving maxillary molar distalization using clear aligner systems. In this review, effectiveness was defined as the amount of distal movement achieved (mm), while predictability was defined as the ratio between achieved and planned movement (%). **Methods**: A systematic review was conducted following the Preferred Reporting Items for Systematic reviews and Meta-Analyses (PRISMA) statement across PubMed-Medline, Scopus, Embase, Web of Science, and Cochrane Handbook databases, with no restrictions on language or publication date. The search was performed up to April 2026. Studies evaluating maxillary molar distalization with clear aligners were screened based on predefined eligibility criteria. **Results**: Of the 1388 articles initially identified, 16 met the inclusion criteria (11 retrospective cohort studies and 5 prospective experimental studies), comprising a total of 461 patients. Reported distalization effectiveness varied widely, ranging from 31.1% to 88.4%. A meta-analysis could not be performed owing to substantial clinical and methodological heterogeneity. **Conclusions**: Clear aligners can achieve maxillary molar distalization; however, the effectiveness decreases as greater distal movement is prescribed, making it unlikely that the clinician’s ideal plan will be fully realized. The use of auxiliary elements, such as Class II elastics, attachments, or skeletal anchorage devices, may reduce anterior proclination and enhance posterior anchorage. Longer aligner-wear intervals (e.g., 14 days) were associated with higher predictability in several of the included studies, although the current evidence is insufficient to establish this interval as definitively superior.

## 1. Introduction

A variety of techniques have been proposed to manage Class II malocclusions in patients of different ages and with diverse anatomical characteristics [1]. When the aim is to correct a dentoskeletal Class II relationship, functional orthopedic treatment is generally considered the option of choice; however, this approach is limited to growing patients and primarily targets the skeletal component. In individuals who have completed growth, maxillary molar distalization represents a viable alternative. This treatment modality is effective for establishing a Class I molar and canine relationship, gaining space, and retracting the maxillary anterior teeth [2].

Since the 1950s, headgear has been the most widely used appliance for maxillary molar distalization. Nevertheless, successful distalization with this approach relies heavily on patient cooperation and adherence to the prescribed intraoral and extraoral devices [3].

The use of fixed multibracket appliances to distalize maxillary molars has been associated with undesirable side effects, including molar tipping and/or loss of anterior anchorage during distalization [4]. In contrast, some authors have suggested that these unwanted movements may be minimized when treating with clear aligners, as the full coverage of the crowns may provide improved vertical and transverse control. A key limitation of this approach, however, is the frequent need for additional anchorage devices to enhance the predictability of the planned distalization [4].

Conversely, other authors argue that the predictability and effectiveness of molar distalization with clear aligners remain inadequate, and that the amount of movement achieved with aligners is inferior to that obtained with multibracket fixed appliances or with temporary skeletal anchorage devices (TSADs) used as adjuncts. One possible explanation is that, unlike Ni-Ti archwires, the force delivered by aligners exhibits exponential stress relaxation within the first hours of wear. Moreover, intraoral degradation of the polymeric material may further decrease the forces exerted during treatment [5,6].

Although most of the current evidence focuses on the Invisalign^®^ system, numerous competing clear aligner systems are now available, employing different materials, thicknesses, and digital planning software. These variations may influence the biomechanics and, consequently, the predictability of tooth movement [5].

Therefore, a clear understanding of the predictability and magnitude of maxillary molar distalization achievable with clear aligners, the optimal aligner change interval for predictable distalization, and the influence of attachments or intermaxillary elastics on treatment outcomes is essential. Such knowledge may help identify the most predictable and effective protocol for maxillary molar distalization with clear aligner systems.

## 2. Materials and Methods

### 2.1. Registration and Focused Question

This systematic review was conducted in accordance with the Preferred Reporting Items for Systematic Reviews and Meta-Analyses (PRISMA statement) and the Cochrane Handbook for Systematic Reviews of Interventions version 6.5 (updated August 2024). Cochrane, 2024. Available from www.cochrane.org/handbook. [7]. The systematic review protocol was previously registered in PROSPERO (Registration ID: CRD42024573061).

The aim was to synthesize the available evidence regarding maxillary molar distalization using clear aligners. The PICO question guiding the review was: “What is the effectiveness of maxillary molar distalization with clear aligners, and which factors influence its predictability?”

Given the heterogeneity in aligner brands, staging protocols, auxiliaries, measurement methods, and timing of outcome assessment described in the following sections, this objective was understood as a synthesis of the available evidence on effectiveness and predictability, rather than an attempt to identify a single, universally superior distalization protocol.

### 2.2. Eligibility Criteria

The inclusion criteria comprised:

Population: Patients undergoing orthodontic treatment with clear aligners requiring maxillary molar distalization.

Intervention: Orthodontic treatment with clear aligners involving maxillary molar distalization.

Comparison: Comparison between planned and achieved tooth movement, and across different treatment-related factors (e.g., aligner change protocols, use of attachments or auxiliaries).

Outcome: Effectiveness (capability of an intervention to produce a desired, beneficial result) and predictability (discrepancy between planned and achieved movement) of maxillary molar distalization and factors influencing the results.

For the purposes of this review, effectiveness was defined as the amount of distal movement achieved, measured in millimeters, reflecting whether clear aligners produced a clinically useful movement; predictability was defined as the ratio between the achieved and the planned (prescribed) distal movement, expressed as a percentage. Although related, these two outcomes are conceptually distinct, and both terms are used according to these definitions throughout the Results, Discussion, and tables. Whenever reported by the original studies, bodily distal movement and distal tipping were considered different types of tooth movement and were described separately.

Controlled clinical trials, randomized clinical trials (RCTs), cohort studies, case–control studies, cross-sectional studies, and multicenter studies were included.

Exclusion criteria included studies not addressing the PICO question, systematic or narrative reviews, case series, case reports, and in vitro investigations.

### 2.3. Search Strategy and Information Sources

An electronic search was performed across five major health science databases: PubMed-Medline, Scopus, Embase, Web of Science, and Cochrane Handbook. Reference lists of the included articles were hand-searched to identify additional eligible studies not detected electronically. The search was conducted up to 1 April 2026, with no restrictions on publication year or language.

The search strategy employed a combination of three MeSH (Medical Subject Headings) terms (“Predictability,” “Distalization,” and “Aligners”) and twelve additional free-text keywords, such as ‘Movement accuracy’, ‘Efficacy’, ‘Molar distalization’, ‘Upper molar distalization’, ‘Maxillary molar distalization’, ‘maxillary arch distalization’, ‘Sequential distalization’, ‘distal movement’, ‘Clear aligner’, ‘clear aligner therapy’, ‘thermoplastic aligner’ and ‘Invisalign’. Terms were combined using Boolean operators (“AND,” “OR”) in predefined groups to ensure a comprehensive search. The complete, database-specific search strategies, including field tags, Boolean operators, filters, and search dates, are reported in Table 1.

### 2.4. Screening Process and Data Collection

All retrieved studies were exported to Mendeley Desktop (version 1.19.5; Mendeley Ltd., Elsevier, https://www.mendeley.com) for the removal of duplicates. Screening followed the PRISMA statement and was performed in sequential phases:

Title screening by two independent reviewers (PB and NZ-M).

Abstract review to assess preliminary relevance.

Full-text evaluation for compliance with inclusion criteria.

Disagreements were resolved by discussion or consultation with a third reviewer (CB-A). The two authors undertook a comprehensive review of the full texts of the articles in question, with a view to ensuring that they met the ideal requirements for entry into the results table. Some articles were excluded from further analysis because they lacked the requisite data for inclusion or did not address the PICO question.

Data extracted from each included study comprised: author, publication year, study design, measurement protocol, sample size, demographic characteristics, aligner brand, treatment characteristics, aligner change interval, outcome variables, and assessed risk of bias.

### 2.5. Risk of Bias and Quality Assessment

The quality of observational studies was assessed by the same investigators independently. The Newcastle–Ottawa Scale (NOS) [8] was used, which evaluates selection, comparability, and outcomes, with a maximum score of nine points. The scale was used for the assessment of cohort, case–control, and cross-sectional studies and comprises eight items distributed across three categories: patient selection, comparability of study groups, and outcomes.

The eight items are set out below:

Selection

1. Representativeness of the exposed cohort.
2. Selection of the unexposed cohort.
3. Verification of exposure.
4. Demonstration that the outcome of interest was not present at the start of the study.

Comparability

5. Comparability of cohorts based on study design or analysis.

Outcomes

6. Assessment of the outcome.
7. Was the follow-up period long enough for the outcomes to occur?
8. Adequacy of cohort follow-up.

Each of these elements is awarded a single star, except for comparability, which may receive a maximum of two stars, resulting in a maximum score of nine stars.

Prospective experimental studies were evaluated using the ROBINS-I tool [9] and the Risk-of-bias plots were generated using the robvis package (version 0.3.0) in R [10] for non-randomized intervention studies. Studies labelled by their original authors as “prospective” were non-randomized prospective clinical studies comparing software-planned and achieved tooth positions, rather than randomized controlled trials; this terminology is used descriptively throughout the review, and risk of bias for these studies was assessed with ROBINS-I rather than a randomized-trial tool. No true cross-sectional studies reporting planned-versus-achieved distalization outcomes were identified during the search, and none were ultimately included in the qualitative synthesis.

The scale comprises seven items, and each item is assigned a value (low, moderate, serious, critical, or no information) based on the extent of compliance with the corresponding item.

The seven items are set out below:

1. Bias in the randomisation process.
2. Allocation concealment.
3. Blinding of participants and staff.
4. Blinding of outcome assessment.
5. Incomplete outcome data.
6. Bias in the selection of reported outcomes.
7. Other sources of bias.

Any discrepancies among the researchers were resolved through consensus, and if necessary, a third researcher (CB-A) was consulted to address any concerns.

Inter- and intra-examiner reliability was assessed using Cohen’s kappa coefficient, with a reported value of 0.88, indicating high reproducibility. Additionally, the GRADEpro GDT (GRADEpro Guideline Development Tool) (https://www.gradepro.org) was used to evaluate the certainty of evidence and classify its quality. The certainty of the evidence was rated for the two main outcomes of this review (achieved distal movement and predictability of maxillary molar distalization) across the domains of risk of bias, inconsistency, indirectness, imprecision, and publication bias, following the GRADE approach.

## 3. Results

### 3.1. Study Selection

The preliminary electronic search identified 1388 references across the five databases: 534 from PubMed, 206 from Scopus, 17 from Embase, 627 from Web of Science, and 4 from Cochrane. One additional study was detected through manual searching. After removing 149 duplicates and excluding 10 records not retrievable, 1229 records remained for title and abstract screening. A total of 1174 studies were subsequently excluded for reasons including unavailable full text, inadequate study design, or not meeting inclusion criteria. The full texts of 55 articles were assessed, of which 39 were excluded: 35 for not adequately addressing the PICO question and 4 for failing to properly evaluate molar distalization. Reasons for exclusion are detailed in Table S1.

**Figure 1:**
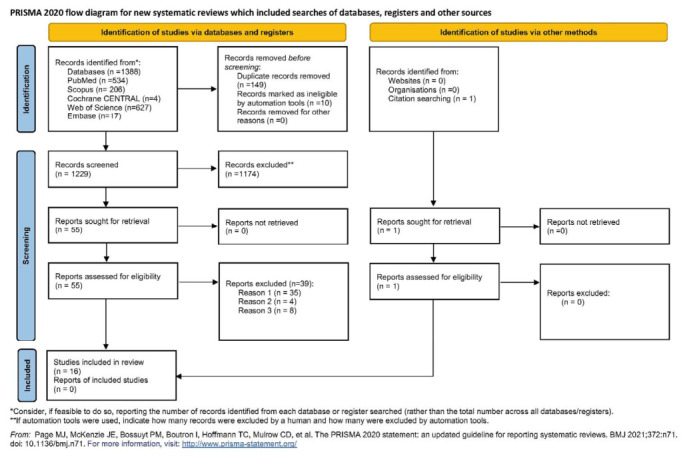
PRISMA 2020 flow diagram [7] for new systematic reviews, which includes searches of databases, registers, and other sources.

Ultimately, 16 studies met all eligibility criteria and were included in the final synthesis. The PRISMA 2020 checklist was followed (Table S2) and the PRISMA 2020 flowchart [7] summarizing the selection process is presented in Figure 1.

### 3.2. Study Characteristics

The variables collected from all the articles included in the present review are compiled in Table 2, Table 3 and Table 4, which separately summarize study characteristics, treatment protocols, and outcomes, in accordance with reviewer recommendations.

The 16 included articles comprised 11 retrospective cohort studies [2,4,6,13,14,16,18,19,20,21,22] and 5 prospective experimental studies [5,11,12,15,17], published between 2014 and 2024. The combined sample consisted of 461 patients: 117 males, 295 females, and 25 participants whose sex was not reported. Participant age ranged from a mean of 36 years [12] to 21.6 years [13], depending on the study.

Most studies employed Invisalign^®^ aligners; two used other commercial systems (Anglealign^®^ [5] and Ordoline^®^ [11]).

Substantial heterogeneity was observed in measurement methods: many studies used digital models (intraoral scans or scanned plaster models), often paired with ClinCheck^®^ or equivalent planning software to predict and evaluate the discrepancies between the actual and predicted movements [2,5,11,12,13,15,16,17,19]. Three studies relied on radiographic evaluation [4,20,21], one used pre- and post-treatment CBCT imaging to observe 3D tooth movements and changes in mandibular position [14], and three applied a combination of CBCT or lateral cephalograms with digital models [6,8,22].

To evaluate the predictability and analyze the differences between planned and actual movements, some studies employed specific software that superimposed the images based on craniofacial structures or reference planes, such as the pterygoid plane, the palatal plane, or the occlusal plane, and created three-dimensional coordinate systems [4,6,14,16,20,21,22]. In contrast, other studies employed the landmark best-fit alignment method [5,12,13,17] proposed by Grünheid et al. [13], the superposition of the palatal rugae area and landmark register [11,15], or other mathematical models [2,18,19] as a means of measurement.

The determination of final records likewise differed: some authors assessed outcomes at the end of the entire treatment [4,6,12,14,17,18,20,21,22], while others evaluated results immediately after the distalization phase [2,5,11,13,16,19]. Only one study [4] included refinements in the final analysis. A single article divided the sample into two groups, taking records before the retraction of the anterior teeth and when the anterior teeth had already retracted by more than 2 mm [15].

Mean treatment duration varied widely. Some studies did not specify whether the reported timeframes pertained to the maxillary molar distalization phase or to the complete orthodontic treatment. Additionally, seven studies did not report mean treatment time [2,5,11,12,14,16,18]. The shortest treatment time was 6.7 months [15], while the longest mean time was 24.3 months [4].

Of the 16 articles included in the present review, five evaluated the predictability of movements in the mesiodistal plane together with other types of movements, such as anterior torque or rotations [2,9,12,17,19]. The remaining articles mainly evaluated the distalization of maxillary molars in Class II cases [4,5,6,11,14,15,16,18,20,21,22].

Distalization protocols were inconsistent. Approaches included: V-shaped sequential distalization [14,16], 50% sequential distalization [5,11,22], two-thirds sequential protocols [4,20], or unspecified “sequential maxillary distalization” [6,21].

Use of auxiliaries also varied. Many studies incorporated Class II elastics [4,5,6,11,15,16,20,21,22], attachments [2,4,5,6,15,16,19,20,21,22], or microscrews [8,16,18,22], while others did not specify whether auxiliaries were used [2,12,13,14,17,19]. All studies instructed patients to wear aligners 22 h per day.

Aligner change intervals ranged from 7 days to 14 days, with one study using 7-day protocols [21], six studies using 14-day protocols [4,11,13,16,18,19], and four using 10-day protocols [5,12,14,15]. One study directly compared 7- and 14-day schedules [17]. Nevertheless, some studies did not report the change interval, or the change rate was unclear [2,6,20,22].

### 3.3. Results of Individual Studies

The primary objective of this analysis was to ascertain the degree of predictability and the effectiveness associated with the maxillary molar distalization movement achieved using aligners. Reported predictability of maxillary molar distalization varied substantially, ranging from 31.1% [6] to 88.4% [19]. Four of the included studies were confirmed to report predictability/effectiveness values at or below 40% [6,16,21,22], whereas the remaining studies for which a percentage outcome was reported showed higher values, including Miao et al. [11] (approximately 53–58%), Cui et al. [14] (83.4–85.1%) up to a maximum of 88.4% (86.9–88.4%) in Simon et al. study [19].

The amount of achieved distalization in the maxillary first molars also exhibited variability across studies, with a minimum distal movement of 0.84 mm [2] and a maximum of 4.86 mm [18]. In the case of the second molars, the distal movement achieved ranged from 0.86 mm [21] to 2.98 mm [14].

Regarding differences between molars, seven studies found no significant difference between the distalization predictability of first and second molars [2,5,15,16,17,19,21]. Four reported slightly greater predictability in second molars [4,11,12,13], and five did not address the comparison [6,14,18,20,22].

One study directly compared clear aligners with fixed appliances, reporting 17% greater effectiveness in distalizing first molars with aligners and noting that bracket-based distalization produced increased molar tipping and reciprocal mesial drift of second molars [18].

To make this synthesis more structured, Table 5 stratifies the reported effectiveness/predictability and achieved-movement values by two key dimensions: timing of outcome assessment and distalization protocol.

### 3.4. Risk of Bias and Quality Assessment

Using the Newcastle–Ottawa Scale [8], most retrospective studies scored 7 or 8 out of 9, indicating generally good quality; one study scored 6 [18] (Table 6).

**Figure 2:**
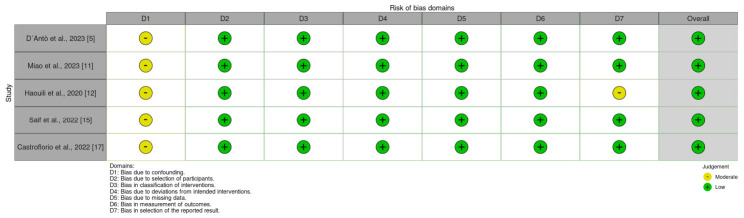
“Traffic light” plots of the domain-level judgements for each individual result according to the ROBINS-I scale [9] (risk of bias of non-randomized studies of interventions) [5,11,12,15,17]. Source: robvis visualization tool (version 0.3.0) in R [10].

**Figure 3:**
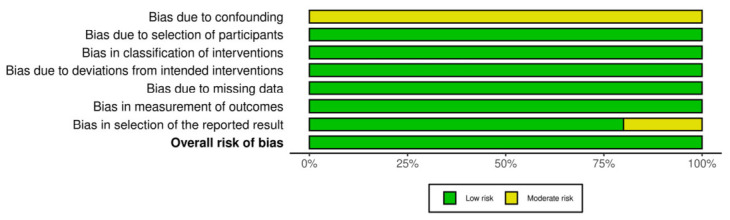
Weighted bar plots of the distribution of risk-of-bias judgements within each bias domain according to ROBINS-I scale [9] (risk of bias of non-randomized studies of interventions). Source: robvis visualization tool (version 0.3.0) in R [10].

The ROBINS-I [9] assessment showed that all prospective studies exhibited an overall low risk of bias, with the confounding domain rated as moderate across all five studies. Risk-of-bias visualizations using the Risk-of-bias plots were generated using the robvis package (version 0.3.0) in R [10] are presented in Figure 2 and Figure 3.

Judgments for each ROBINS-I domain were made at the outcome level (achieved distalization/predictability) rather than at the study level as a whole. Confounding was rated as moderate in all five prospective studies because malocclusion severity, amount of prescribed distalization, use of attachments, elastics, or miniscrews, third-molar status, and operator planning could not be fully controlled or adjusted for in any of the included designs; this factor, together with incomplete or unclear compliance monitoring, prevented an overall “low risk” rating from being interpreted as equivalent to the certainty achievable in a randomized design. For the retrospective cohort studies, Newcastle–Ottawa Scale scores of 7–8 out of 9 indicate adequate reporting of selection, comparability, and outcome ascertainment, but, as recommended, these scores should not be interpreted as excluding residual confounding (e.g., by case severity, prescribed movement, or auxiliary use) or clinically relevant heterogeneity between studies, which remained substantial for both effectiveness and predictability outcomes.

### 3.5. Certainty of the Evidence (GRADE)

The certainty of the evidence for the two main outcomes of this review—achieved maxillary molar distalization (mm) and predictability of maxillary molar distalization (%)—was assessed using the GRADE approach (GRADEpro GDT). Both outcomes were rated down for risk of bias (non-randomized designs with moderate confounding), inconsistency (wide variability in reported values), indirectness (heterogeneous aligner systems, staging protocols, and outcome timing), and imprecision (small samples and inconsistently reported dispersion measures), resulting in an overall certainty of the evidence rated as very low for both outcomes. The Summary-of-Findings table is presented in Table 7.

## 4. Discussion

Maxillary molar distalization has become an increasingly common strategy for correcting Class II malocclusions in adult patients [16]. However, this movement is often associated with undesirable side effects, such as distal tipping of the molars or unwanted extrusion [4]. The introduction of clear aligner therapy has represented a significant advancement in orthodontics, as aligners fully envelop the teeth and may therefore provide enhanced vertical and transverse control compared with traditional fixed appliances [4,13].

In recent years, growing interest has focused on understanding how maxillary molar distalization can be optimized with aligners. The present systematic review aimed to identify the effectiveness of maxillary molar distalization with clear aligners and the factors that influence its predictability.

Among the 16 studies included, five had small sample sizes (fewer than 20 participants), a limitation that can reduce the external validity of individual findings [5,6,14,20,21]. Overall, the combined sample of 461 participants was relatively homogeneous, with mean ages below 40 years and only three studies including adolescents or growing patients [2,15,19]. The studies by Simon et al. [2,19] included patients aged 13 years and older, while Saif et al. [15] included participants aged 17 years and older.

Nine of the studies [4,5,11,14,15,17,18,21,22] included only participants with missing or previously extracted upper third molars, while the remaining seven did not specify third-molar presence [2,6,12,13,16,19,20]. Most studies used the Invisalign^®^ system, with only two investigating other aligner brands, thereby increasing the consistency of the dataset [5,11].

### 4.1. Variability in Measurement Methods

A high level of methodological heterogeneity was found, particularly in measurement techniques. Most studies used digital pre- and post-treatment models created from intraoral scans or digitized plaster models, often evaluated through ClinCheck^®^ or similar software [2,5,11,12,13,15,16,17,19]. Others relied on radiographs [4,20,21] or CBCT [14], while three employed a combined radiographic-digital approach. The use of radiographs alone may introduce inaccuracies in measurement due to potential magnification or distortion, particularly in the case of 2D X-rays. To mitigate potential methodological inconsistencies, these three studies employed a combined approach, integrating lateral teleradiographs or CBCT with pre- and post-treatment digital models [6,18,22].

Superimposition methods also differed widely, using either craniofacial reference planes, palatal rugae, landmark best-fit alignment, or mathematical models. Such variations likely contributed to inconsistencies in reported distalization accuracy.

### 4.2. Aligner Change Protocols

Aligner wear schedules varied across studies, with many employing 14-day [4,11,13,16,18,19] or 10-day intervals [5,12,14,15], and one using a 7-day interval [21]. In the study conducted by Castroflorio et al. [17], the participants were divided into three groups based on the frequency of aligner changes: 7, 10, and 14 days. The authors concluded that changing the aligner every 14 days resulted in a 12% reduction in the lack of correction compared with changing every seven days. Similarly, Al-Nadawi et al. [23] discovered that the 14-day use protocol resulted in significantly enhanced precision in subsequent tooth movements when compared with the 7- and 10-day protocols. Furthermore, Linjawi et al. [24] demonstrated that aligner or attachment misalignment was less after 15 days of wear compared with the 3-, 7-, and 10-day groups. In a recently published systematic review, Monisha et al. [25] concluded that the 7-day, 10-day, and 14-day protocols did not demonstrate any significant difference in efficacy regarding orthodontic tooth movement, except in the case of certain movements, where superior outcomes were observed with the 14-day protocol. Accordingly, the selection of the activation schedule might be contingent upon several factors, including the complexity of the case, patient comfort, and the orthodontist’s treatment philosophy.

### 4.3. Predictability of Maxillary Molar Distalization

The studies demonstrated that clear aligners could achieve distal translational movements averaging around 2–3 mm, consistent with prior literature [4]. The studies conducted by Simon et al. [2,19] determined that maxillary molar distalization using aligners resulted in an average distal movement of 2.6 mm and an effectiveness of 86.9% without the support of an attachment. However, Rossini et al. [26] deemed the methodology of this study to be of limited quality and recommended that the results should be interpreted with caution. As Hmud and Alamri [27] have observed, although the literature indicates that 2–3 mm of molar distalization is feasible, a distinction between bodily movement and the backward molar tip should be made and investigated further.

In contrast, D’Antò et al. [5] reported a predictability of 79.9% for the distalization of the second molar, with a mean achieved distal displacement of 1.5 mm. It should be noted that precision is inversely proportional to the amount of distalization to be achieved.

In opposition to the preceding authors, Li et al. [16] attained a predictability of merely 31.5% for the distalization of the first molar and 35.63% for the second molar. Of the predicted 2.5 mm, only a total of 0.88 mm for the first molar and 1.10 mm for the second molar were achieved. Some authors have proposed that this discrepancy may be attributed to the reduction in aligner length following distalization of the maxillary molars, which was implemented to retract anterior teeth. The reduction in aligner length results in the generation of a mesial force by the aligners towards the maxillary molars, which in turn causes mesial movement of the maxillary molars [16,26,28,29]. In a second study group, the previous authors [16] maintained the length of the aligners, which significantly reduced the anchorage loss of the distalized molars. This resulted in a predictability of 36.48% for the distalization of the first molar and 41.94% for the second molar.

### 4.4. Timing of Outcome Assessment

When comparing studies according to the timing of outcome assessment, an important distinction emerges between those that evaluated distalization immediately after the distalization phase and those that assessed outcomes at the end of comprehensive treatment. Studies ending at the distalization phase generally reported a wide range of predictability values, from relatively low values such as 36.48–41.94% from Li et al. [16] or 36.2–43.9% from Liu et al. [22] to higher values such as 79.9% from D’Anto et al. [5] and 86.9–88.4% from Simon et al. [2,19].

Studies evaluating outcomes at the end of total treatment also tended to show variable predictability values, from 35.39–40.11% in Mamani et al. [21] to 83.44–85.14% in Cui et al. [14], partly because subsequent treatment stages, anterior retraction, settling, or refinement phases may alter the final position of the distalized molars.

However, measurements taken immediately after distalization may better reflect the actual efficiency of the distalization protocol itself, whereas end-of-treatment records reflect the combined effect of the entire orthodontic sequence.

This methodological difference is clinically relevant because it may partly explain the considerable heterogeneity observed across studies. Articles ending after the distalization phase provide a more direct estimate of how much of the programmed distal movement was truly expressed by the aligners, with less interference from later biomechanical steps. By contrast, studies assessing patients only at the end of treatment may underestimate or obscure the true distalization achieved during the active distalization phase, since molar position can be modified by anchorage loss, anterior retraction mechanics, occlusal settling, or refinement aligners. Consequently, direct comparison of predictability percentages across studies should be interpreted with caution unless the timing of measurement is clearly standardized.

### 4.5. Role of Attachments and Auxiliaries

The majority of the authors employed auxiliaries, including those recommended by the manufacturer or optimized attachments [2,4,5,6,15,16,19,20,21,22], microscrews [8,16,18,22], or Class II elastics [4,5,6,11,15,16,20,21,22] with the objective of enhancing the predictability of the movements.

Most authors supported the use of attachments, as they may facilitate maxillary molar distalization and help counteract unwanted tipping. However, some studies [2,19] asserted that there were no notable distinctions between the performance of maxillary molar distalization with or without the use of attachments or auxiliaries. Ravera et al. [4] indicated in their study that rectangular and vertical attachments located on the buccal side of the distalized molars are required to create sufficient reciprocal force to oppose the tilting movement. Other studies have also demonstrated that vertical rectangle-shaped attachments are the most efficient in counteracting the distal tipping effect of maxillary molars [2,4,15,19]. In a recent study, Khursheed Alam et al. [30] concluded that attachments can play a significant role in enhancing specific orthodontic movements. One strategy to enhance posterior anchorage is to incorporate attachments on a larger number of teeth, ranging from the canine to the second molar.

As mentioned above, during molar distalization with aligners, loss of anchorage is a common occurrence, particularly in the incisor area. This is due to the reciprocal reaction force exerted by the teeth in response to the distalization force [31]. Furthermore, in certain instances, the degree of lack of movement and lack of predictability reaches nearly 40–50%, as documented by researchers such as Castroflorio et al. [17] or Saif et al. [15]. Some studies included in the present work have employed Class II elastics during the distalization phase of the maxillary molars or during the retraction of the anterior teeth, with the objective of circumventing these adverse effects [4,5,6,11,15,16,20,21,22].

Some authors have stated that the use of Class II elastics is an effective method for preventing uncontrolled inclination of anterior teeth and maintaining maximum contact of the aligner with the teeth [4,5,15,22]. In contrast, other studies have indicated that the use of Class II elastics is inadequate for preventing anchorage loss and have proposed the use of microscrews to achieve stronger anchorage directly to the molars or between premolars and molars [8,16]. However, the study by Liu et al. [22] compared the use of Class II elastics and microscrews during the distalization phase and found no significant differences between the two groups. In both groups, the achieved distalization of the posterior teeth was significantly less than the predicted amount, with an efficacy ranging from 36.2% to 43.9%. The heterogeneity observed by the authors of the present study between studies regarding the protocols of Class II elastics may introduce bias when identifying the primary risk factors for anchorage loss, as this adverse effect could manifest at any stage of the tooth movement.

### 4.6. Distalization Protocols

Another factor that may exert a significant influence on the predictability of the movements is the distalization protocol. The sequential distalization protocol restricts the separation between the distalized teeth, thereby enhancing the aesthetic quality and ensuring optimal contact between the aligners and the teeth, while simultaneously reducing the flexibility of the plastic material. This may result in a reduction in the unwanted tilting of the incisors, as evidenced clinically by an increased overbite and loss of palatal root torque [4,32]. While some authors employed a V pattern [14,16] or the 50% distalizing protocol [5,11,22], other authors utilized the two-thirds protocol [4,20], and some used the term “sequential maxillary distalization” without specifying the protocol [6,21]. These differences in the type of distalization may also account for the considerable variability in the reported predictability percentages or millimeters of distalization found in the literature. Regardless of the year of publication of the studies, the authors continue to report different protocols. The literature would benefit from controlled comparative studies evaluating the biomechanics and predictability of each protocol.

### 4.7. Limitations of the Existing Literature

Key limitations identified by the review include substantial heterogeneity in sample sizes, distalization protocols, and aligner wear schedules; inconsistent measurement methodologies and superimposition techniques; differences in timing of post-treatment assessments; and limited reporting on patient compliance, despite instructions to wear aligners 22 h/day.

In terms of methodology, the authors identified significant methodological heterogeneity, with studies employing superimposed digital models [2,5,11,12,13,15,16,17,19] and others utilizing radiographic records [4,14,20,21]. Although cephalometric-only studies do not allow assessment of predictability, they were included to evaluate treatment effectiveness; however, this represents a methodological limitation of the present review.

Furthermore, the superimposition method differed across studies. Some employed craniofacial structures or reference planes [4,6,14,16,20,21,22] to assess discrepancies between planned and actual movements, while others utilized the area of the palatal rugae [11,15], best-fit alignment methods [5,12,13,17], or alternative mathematical models [2,18,19].

Such discrepancies may result in variations in the reported results between different studies. It is therefore recommended that both techniques be used for future studies, as exemplified by the work of Li et al. [6], which used pre- and post-treatment CBCTs to observe 3D tooth movements and changes in jaw position, together with the use of overlay digital models to evaluate the effectiveness of movements.

As detailed above in the dedicated subsection on the Timing of Outcome Assessment, the point at which distalization outcomes were recorded—immediately after the distalization phase versus at the end of comprehensive treatment—constitutes a key, previously discussed source of heterogeneity among the included studies and should be borne in mind when interpreting the pooled range of predictability values reported below.

A formal meta-analysis of studies evaluating outcomes at the end of the distalization phase was considered; however, it was not performed because of substantial methodological and statistical heterogeneity across the included studies. The available articles differed in outcome definition, reporting format, units of analysis, and measurement timing. Some studies reported distalization in millimeters, others reported percentage of predictability, and several presented cusp-specific or tooth-specific measurements without providing sufficient dispersion data for pooling. In addition, standard deviations or confidence intervals were inconsistently reported, preventing robust quantitative aggregation.

Nevertheless, a descriptive quantitative synthesis of the studies ending at the distalization phase suggests marked variability in treatment performance. Reported predictability ranged from approximately 31.5% to 88.4%, while achieved distalization generally varied between about 0.9 mm and 2.6 mm. Higher predictability values were reported in some studies using carefully staged protocols and controlled distalization mechanics, whereas lower values were observed in studies involving greater prescribed movements or anchorage-demanding protocols. These findings indicate that clear aligner distalization is feasible, but highly sensitive to protocol design, measurement method, and timing of assessment.

The lack of standardization across studies highlights the need for future research using uniform outcome measures, clearly defined distalization endpoints, and complete statistical reporting. Prospective controlled studies should report planned and achieved movement separately for first and second molars, together with standard deviations and sample sizes, to allow valid pooled estimates in future meta-analyses.

Despite this limitation, both types of studies were included to avoid excluding relevant clinical evidence and to provide a broader overview of treatment outcomes. Therefore, the timing of measurements should be carefully considered when interpreting the results of this review.

A further limitation of the review—and, in the authors’ view, a major source of uncertainty rather than a minor caveat—is that, although all articles instructed patients to wear the aligners for 22 h per day, as recommended by the manufacturer, only a few articles described how compliance was verified, and typically only through verbal confirmation from participants after each appointment [4,11,12,15,16]. Because wear time directly determines the force and duration of loading actually delivered to the maxillary molars, undetected variability in compliance may account for a substantial part of the observed variability in both achieved distalization and predictability, independent of aligner system, staging protocol, or auxiliary use. One study included non-compliance performance among its exclusion criteria [21], whereas the remaining studies did not specify or report data on this variable [2,5,6,13,14,17,18,19,20,22]. Some manufacturers include features named “compliance indicators”. It should be noted, however, that these features are only available in protocols and products designed for teenage patients.

These factors likely contribute to the large variability in distalization predictability across studies. Future research should standardize protocols, incorporate larger samples, and employ precise, reproducible measurement strategies.

## 5. Conclusions

### 5.1. Accuracy of Maxillary Molar Distalization

The predictability of distalization for first and second maxillary molars ranged from 31.1% to 88.4%, with an overall average movement of approximately 2 mm. However, it is unlikely that the clinician’s ideal prescription will be fully achieved, and additional adjustments or refinement stages are often required.

### 5.2. Lack of Consensus on Optimal Distalization Protocol

There is currently no agreement in the literature regarding the most effective sequential distalization protocol. Comparative studies designed to evaluate different staging approaches are needed to determine which protocol yields the most predictable outcomes.

### 5.3. Role of Auxiliaries

Class II elastics, miniscrews, and attachments should be regarded as potentially useful adjuncts, rather than as uniformly proven solutions across all protocols and patients. When used, Class II elastics or miniscrews during maxillary molar distalization with aligners may help limit uncontrolled proclination of the anterior teeth and enhance posterior anchorage, and rectangular or vertical attachments placed on the buccal surfaces of molars have been associated with reduced distal tipping forces during treatment; however, the comparative superiority of any single auxiliary strategy has not been established.

### 5.4. Aligner Change Intervals

Longer aligner-change intervals (e.g., 14 days) were associated with increased movement accuracy and improved predictability of maxillary molar distalization in several of the included studies. However, only one study directly compared 7-, 10-, and 14-day intervals specifically for maxillary molar distalization, and aligner change interval is confounded by case complexity, staging protocol, aligner material, attachment design, patient compliance, and prescribed movement per aligner. The available evidence therefore suggests, rather than confirms, a possible advantage of longer wear intervals, and is not sufficient to support a definitive clinical recommendation of 14 days as superior specifically for maxillary molar distalization.

## Figures and Tables

**Table 1:** Search strategy for identifying studies in electronic primary databases.

Database/Source	Search Strategy, Filters, and Search Date
PubMed-Medline (searched via PubMed.gov)	(“predictability” [Title/Abstract] OR “movement accuracy” [Title/Abstract] OR “efficacy” [Title/Abstract]) AND (“distalization” [Title/Abstract] OR “molar distalization” [Title/Abstract] OR “upper molar distalization” [Title/Abstract] OR “maxillary molar distalization” [Title/Abstract] OR “sequential distalization” [Title/Abstract] OR “maxillary arch distalization” [Title/Abstract] OR “distal movement” [Title/Abstract]) AND (“aligner” [Title/Abstract] OR “clear aligner” [Title/Abstract] OR “clear aligner therapy” [Title/Abstract] OR “thermoplastic aligner” [Title/Abstract] OR “invisalign” [Title/Abstract]) Filters: none (all languages, all publication dates). Search date: up to 1 April 2026. Records retrieved: 534.
Scopus	TITLE-ABS-KEY ((“predictability” OR “movement accuracy” OR “efficacy”) AND (“distalization” OR “molar distalization” OR “upper molar distalization” OR “maxillary molar distalization” OR “sequential distalization” OR “maxillary arch distalization” OR “distal movement”) AND (“aligner” OR “clear aligner” OR “clear aligner therapy” OR “thermoplastic aligner” OR “invisalign”)) Filters: none applied. Search date: up to 1 April 2026. Records retrieved: 206.
Embase (embase.com)	(‘predictability’/exp OR ‘movement accuracy’:ti,ab OR ‘efficacy’/exp) AND (‘tooth distalization’/exp OR ‘distalization’:ti,ab OR ‘molar distalization’:ti,ab OR ‘upper molar distalization’:ti,ab OR ‘maxillary molar distalization’:ti,ab OR ‘sequential distalization’:ti,ab OR ‘maxillary arch distalization’:ti,ab OR ‘distal movement’:ti,ab) AND (‘clear aligner’/exp OR ‘aligner’:ti,ab OR ‘clear aligner’:ti,ab OR ‘clear aligner therapy’:ti,ab OR ‘thermoplastic aligner’:ti,ab OR ‘invisalign’:ti,ab) Filters: none applied. Search date: up to 1 April 2026. Records retrieved: 17.
Web of Science (Core Collection)	TS = ((“predictability” OR “movement accuracy” OR “efficacy”) AND (“distalization” OR “molar distalization” OR “upper molar distalization” OR “maxillary molar distalization” OR “sequential distalization” OR “maxillary arch distalization” OR “distal movement”) AND (“aligner” OR “clear aligner” OR “clear aligner therapy” OR “thermoplastic aligner” OR “invisalign”)) Filters: none applied. Search date: up to 1 April 2026. Records retrieved: 627.
Cochrane Library (CENTRAL, Cochrane Handbook line-by-line strategy)	#1 (aligner OR “clear aligner” OR “clear aligner therapy” OR “thermoplastic aligner” OR invisalign):ti,ab,kw #2 (distalization OR “molar distalization” OR “upper molar distalization” OR “maxillary molar distalization” OR “sequential distalization” OR “maxillary arch distalization” OR “distal movement”):ti,ab,kw #3 (predictability OR “movement accuracy” OR efficacy):ti,ab,kw #4 #1 AND #2 AND #3 Filters: none applied. Search date: up to 1 April 2026. Records retrieved: 4.
Hand-search	Reference lists of all included articles were manually screened to identify additional eligible studies not detected electronically. Records retrieved: 1.

**Table 2:** Characteristics of the included studies (study design, sample, and measurement protocol). m: men; w: women; N: sample size; y: year.

Author/Year	Study Design	Sample Size, Age, Gender	Aligner System	Records/Measurement Protocol
Simon et al., 2014 [2]	Retrospective cohort study	N = 26; 11 m, 15 w; mean age 32.9 ± 16.3 y (range 13–72)	Invisalign^®^; >1.5 mm of molar distalization required	Plaster and resin models; pre-treatment, planned (ClinCheck^®^) and end-of-distalization records
Ravera et al., 2016 [4]	Retrospective multicenter study	N = 20; 9 m, 11 w; mean age 29.73 ± 6.89 y	Invisalign^®^; sequential distalization protocol “two-thirds”	Lateral cephalograms at T0 (beginning) and T1 (end of treatment)
D’Antò et al., 2023 [5]	Prospective experimental study	N = 16; 4 m, 12 w; mean age 25.7 ± 8.8 y (range 18–45.5)	Ordoline^®^ aligners	Intraoral STL scans; pre-treatment, planned (set-up) and final records (end of distalization phase)
Lin et al., 2023 [6]	Retrospective cohort study	N = 7; mean age 26.64 ± 3.02 y (range 23.1–31.5)	Invisalign^®^; upper molar distalization with Class II elastics	CBCT + intraoral STL scans; pre-treatment, planned (ClinCheck^®^) and final records (end of treatment)
Miao et al., 2023 [11]	Prospective experimental study	N = 22 molars; 7 m, 15 w; mean age 25.94 y	Angelalign^®^ (EA Medical Instruments©)	Intraoral STL scans; pre-treatment, planned (set-up) and final records (end of distalization phase)
Haouili et al., 2020 [12]	Prospective experimental study	N = 38; 13 m, 25 w; mean age 36 y	Invisalign^®^; different types of movements	Intraoral STL scans; pre-treatment, planned (ClinCheck^®^) and final records (end of treatment)
Grünheid et al., 2017 [13]	Retrospective cohort study	N = 30; 13 m, 17 w; mean age 21.6 ± 9.8 y	Invisalign^®^; different types of movements	Scanned plaster cast models; pre-treatment, planned (ClinCheck^®^) and end-of-distalization records
Cui et al., 2022 [14]	Retrospective cohort study	N = 18; gender not specified; mean age 27.8 ± 5.38 y (range 18–38)	Invisalign^®^; V-pattern bilateral maxillary first-molar distalization	3D CBCT scans; pre-treatment, planned (ClinCheck^®^) and final records (end of treatment)
Saif et al., 2022 [15]	Prospective experimental study	N = 38; 4 m, 34 w; mean age 25.4 y (range 17–39)	Invisalign^®^; maxillary molar distalization without Class II elastics	Intraoral STL scans; pre-treatment, planned (ClinCheck^®^) and final records (end of distalization phase)
Li et al., 2023 [16]	Retrospective cohort study	N = 43; 5 m, 38 w; mean age 28.15 ± 6.94 y	Invisalign^®^; maxillary sequential distalization with V-pattern; 2-week protocol	Scanned plaster cast models; pre-treatment, planned (ClinCheck^®^) and final records (end of distalization)
Castroflorio et al., 2023 [17]	Prospective experimental study	N = 79; 23 m, 56 w; mean age 30.8 ± 12 y	Invisalign^®^; different types of movements	Intraoral STL scans; pre-treatment, planned (ClinCheck^®^) and final records (end of treatment)
Chen et al., 2023 [18]	Retrospective observational case–control study	N = 40; 20 m, 20 w; mean age 28.5 ± 1.5 y (range 18–35)	Invisalign^®^ (Group 2) vs. multibracket fixed appliances + microimplants (Group 1)	Plaster models, digital models and radiographs; pre-treatment, planned (set-up) and final records (end of treatment)
Simon et al., 2014 [19]	Retrospective cohort study	N = 30; 11 m, 19 w; mean age 32.9 ± 16.3 y (range 13–72)	Invisalign^®^; molar distalization with and without attachments	Scanned plaster cast models; pre-treatment, planned (ClinCheck^®^) and end-of-distalization records
Caruso et al., 2019 [20]	Retrospective cohort study	N = 10; 2 m, 8 w; mean age 22.7 ± 5.3 y	Invisalign^®^; sequential distalization protocol “two-thirds”	Lateral cephalograms at T0 (beginning) and T1 (end of treatment)
Mamani et al., 2024 [21]	Retrospective cohort study	N = 14; mean age 33.61 ± 8.57 y (range 20–49)	Invisalign^®^; sequential molar distalization with Class II elastics	Lateral cephalograms at T0 (beginning) and T1 (end of treatment); planned movement assessed with ClinCheck^®^
Liu et al., 2024 [22]	Retrospective multicenter study	N = 30; two groups: N = 17 miniscrew group (mean age 26.7 ± 5.2 y), N = 13 elastics group (mean age 30.4 ± 9.3 y)	Invisalign^®^; maxillary sequential distalization protocol “50%”	CBCT + intraoral STL scans; pre-treatment, planned (ClinCheck^®^) and final records (end of treatment)

**Table 3:** Treatment protocols of the included studies (staging protocol, attachments/auxiliaries, third-molar status, and aligner change interval). NR: not reported/not specified in the original publication.

Author/Year	Distalization/Staging Protocol	Attachments/Auxiliaries	Third-Molar Status	Aligner Change Interval, Wear Time, Treatment Duration
Simon et al., 2014 [2]	Molar distalization ≥ 1.5 mm with horizontal beveled gingival attachment	Horizontal beveled gingival attachment	NR	Not specified
Ravera et al., 2016 [4]	Sequential “two-thirds” distalization protocol; rectangular vertical attachments in molars/premolars	Rectangular vertical attachments in molars and premolars; Class II elastics to reinforce anchorage during anterior retraction	Not present or previously extracted	Aligner change every 14 days; wear time 22 h/day; average treatment time 24.3 ± 4.2 months
D’Antò et al., 2023 [5]	Distalizing staging protocol of 50%, horizontal rectangular attachments in 1st/2nd molars	Rectangular attachments on 1st/2nd molars; Class II elastics all day	Not present or previously extracted	Aligner change every 10 days; wear time 22 h/day
Lin et al., 2023 [6]	Upper molar distalization with Class II elastics; unspecified “sequential maxillary distalization”	Class II elastics	NR, not specified	Aligner change/wear time not specified; average treatment time 13.78 ± 8 months
Miao et al., 2023 [11]	Sequential molar distalization protocol of 50%	Microimplants between 2nd premolar and 1st molar to prevent anchorage loss	Not present or previously extracted	Aligner change every 14 days; wear time 22 h/day
Haouili et al., 2020 [12]	Not specified as a distalization-specific staging protocol	Not specified	NR	Aligner change every 10 days; wear time 22 h/day
Grünheid et al., 2017 [13]	Not specified as a distalization-specific staging protocol	Not specified	NR	Aligner change every 14 days; wear time 22 h/day; average treatment time 11 ± 4 months
Cui et al., 2022 [14]	V-pattern sequential distalization for bilateral maxillary first molars	Not specified	Not present or previously extracted	Aligner change every 10 days; wear time 22 h/day
Saif et al., 2022 [15]	Maxillary molar distalization without Class II intermaxillary elastics; auxiliaries other than attachments	Auxiliaries other than attachments; no Class II elastics	Not present or previously extracted	Aligner change every 10 days; wear time 22 h/day; average time to achieve distalization 6.7 months
Li et al., 2023 [16]	Maxillary sequential distalization with V-pattern (2-week aligner-change protocol); Class II elastics or miniscrews to reinforce anchorage	Class II elastics or miniscrews (manufacturer-recommended attachments)	NR, not specified	Aligner change every 14 days; wear time 22 h/day
Castroflorio et al., 2023 [17]	Comparison of 7-, 10- and 14-day aligner-change groups (treatment design)	Not specified	Not present or previously extracted	Aligner change every 7–14 days (compared groups); wear time 22 h/day
Chen et al., 2023 [18]	Group 1: fixed appliances + microimplants; Group 2: clear aligners	Microimplants (both groups)	Not present or previously extracted	Aligner change every 14 days
Simon et al., 2014 [19]	Molar distalization with (a) and without (b) attachment support	Rectangular attachment (subgroup a) vs. no attachment (subgroup b)	NR	Aligner change every 14 days; wear time 22 h/day
Caruso et al., 2019 [20]	Sequential “two-thirds” distalization protocol; vertical rectangular attachments in upper molars/premolars	Class II elastics; vertical rectangular attachments in upper molars and premolars	NR	Aligner change/wear time not specified; average treatment time 1.9 ± 0.5 years
Mamani et al., 2024 [21]	Sequential maxillary molar distalization with Class II elastics	Class II elastics	Not present or previously extracted	Aligner change every 7 days; wear time 22 h/day
Liu et al., 2024 [22]	Maxillary sequential distalization “50%” protocol; miniscrew anchorage vs. Class II elastics	Miniscrews (one group) vs. Class II elastics (other group)	Not present or previously extracted	Aligner change every 7–14 days; wear time 22 h/day

**Table 4:** Outcomes of the included studies (planned vs. achieved movement, predictability, molar comparison, and key results). M: molar; Percentages in Table 4 that were not explicitly reported by the original authors were calculated by the review team as achieved/planned movement × 100 and are indicated as such.

Author/Year	Timing of Assessment	Planned vs. Achieved Movement	Predictability (%)	1st vs. 2nd Molar Comparison	Key Results/Adverse Effects
Simon et al., 2014 [2]	End of distalization phase	Predicted distalization 1.5–3.9 mm; mean achieved 2.6 mm	86.9% (without attachment support), reported jointly with Simon et al. [19]	Not significantly different between molars	Average real movement 2.6 mm; movement of upper molars was the most effective movement
Ravera et al., 2016 [4]	End of comprehensive treatment	1st M distalized 2.25 mm; 2nd M distalized 2.52 mm	Not reported as a single percentage	Second molar showed slightly greater distal movement than first	Distal movement without significant tilting (*p* = 0.27 and *p* = 0.056) or vertical movement
D’Antò et al., 2023 [5]	End of distalization phase	Prescribed distal movement 2 mm; achieved 1.5 mm	79.9% (2nd molar)	Not addressed	Significant differences between prescribed and achieved movement
Lin et al., 2023 [6]	End of comprehensive treatment	1st M distobuccal cusp achieved 0.84 ± 0.6 mm	37.2% (mesiopalatal cusp) to 56.1% (distobuccal cusp)	Not addressed	Achieved 1st M distalization significantly less than planned; treatment accuracy varied by cusp
Miao et al., 2023 [11]	End of distalization phase	1st M: 1.25 ± 0.79 mm achieved vs. higher predicted; 2nd M: 1.41 ± 1.00 mm achieved vs. higher predicted (both *p* < 0.001)	~57.6% (1st M) and ~53.0% (2nd M), calculated from reported means	Deviational movements between 1st M with/without attachments differed (*p* = 0.011)	Achieved distalization significantly different from predicted values in both groups
Haouili et al., 2020 [12]	End of comprehensive treatment	Not reported in mm	62.4% (2nd M distal crown tip) vs. 58.4% (1st M)	2nd molar more accurate than 1st molar	Distal crown tip movement of 2nd M more accurate than 1st M
Grünheid et al., 2017 [13]	End of distalization phase	Difference for 1st M: 0.27 ± 0.30 mm (CI 0.14–0.41); 2nd M: 0.07 ± 0.81 mm (CI 0.07–0.20)	Not reported as a single percentage (landmark best-fit method)	Not significantly different between molars	Significant differences between intended and achieved tooth positions
Cui et al., 2022 [14]	End of comprehensive treatment	1st M distalization range 2.57 ± 1.15 mm (*p* < 0.01); 2nd M 2.98 ± 1.84 mm (*p* < 0.01)	83.44% (1st M) and 85.14% (2nd M)	Not significantly different between molars	No obvious vertical movement or tilt (*p* < 0.05)
Saif et al., 2022 [15]	End of distalization phase	1st M: prescribed 2.39 ± 1.03 mm, achieved 1.81 ± 0.84 mm; 2nd M: prescribed 2.56 ± 1.06 mm, achieved 1.85 ± 0.88 mm	~75.7% (1st M) and ~72.3% (2nd M), calculated from reported means	Not significantly different between 1st and 2nd molars	Significant differences between prescribed and achieved movement; correlation between distal movement and anchorage loss of incisors
Li et al., 2023 [16]	End of distalization phase	1st M: 0.882 ± 0.8299 mm achieved vs. 2.418 ± 1.1908 mm planned (*p* < 0.001); 2nd M: 1.108 ± 0.9661 mm achieved vs. 2.642 ± 1.2279 mm planned (*p* < 0.001)	36.48% (1st M) and 41.94% (2nd M) overall; retraction group 31.5%/35.63%; non-retraction group 48.14%/52.51%	Not significantly different between molars	Significant difference between retraction and non-retraction subgroups
Castroflorio et al., 2023 [17]	End of comprehensive treatment	1st M predicted movement 0.49 ± 0.74 mm, lack of movement 0.21 ± 0.26 mm; 2nd M predicted 0.45 ± 0.78 mm, lack of movement 0.21 ± 0.20 mm	Not reported as a single percentage; 14-day interval associated with 12% less lack of correction than 7-day interval	Not significantly different between molars	Lack of movement significant for all movements (*p* < 0.01); 14-day protocol reduced lack of correction vs. 7-day protocol
Chen et al., 2023 [18]	End of comprehensive treatment	G1: 4.78 ± 0.57 mm total distalization; G2 (aligners): 4.86 ± 0.27 mm total distalization	Comparative: 17% greater efficacy reported for aligners (G2) vs. fixed appliances (G1); 19 fewer days to distalize 1st M in G2	Not addressed for 1st vs. 2nd molar within aligner group	Fixed-appliance distalization associated with greater molar tipping and mesial drift of 2nd molars
Simon et al., 2014 [19]	End of distalization phase	Not reported in absolute mm in this subgroup analysis	88.4% with attachment (SD 0.2); 86.9% without attachment (SD 0.16)	Distalization of upper molars was the most effective movement regardless of attachment use	High accuracy of molar distalization irrespective of attachment use
Caruso et al., 2019 [20]	End of comprehensive treatment	Not reported in mm	Not reported as a percentage	Not addressed	Significant distal movement of upper molars (*p* < 0.01) without distal tipping, confirming bodily movement with vertical control
Mamani et al., 2024 [21]	End of comprehensive treatment	1st M: 0.91 ± 0.28 mm achieved vs. 2.23 ± 0.27 mm planned; 2nd M: 0.86 ± 0.25 mm achieved vs. 2.43 ± 0.53 mm planned	40.11% (1st M) and 35.39% (2nd M)	Second molar showed slightly lower predictability than first	Actual distalization markedly lower than planned for both molars
Liu et al., 2024 [22]	End of comprehensive treatment	Miniscrew group 1st M: 1.21 ± 1.15 mm vs. 3.34 ± 0.90 mm planned; 2nd M: 1.56 ± 1.28 mm vs. 3.66 ± 0.88 mm; Elastics group 1st M: 1.40 ± 0.86 mm vs. 3.36 ± 0.79 mm; 2nd M: 1.55 ± 0.83 mm vs. 3.59 ± 0.74 mm (all *p* < 0.001)	36.2–43.9% (efficiency of maxillary arch distalization); no significant difference between miniscrews and elastics	No significant difference between 1st and 2nd molar performance	Achieved distalization significantly less than predicted in both groups; no significant difference between miniscrew and elastics anchorage

**Table 5:** Descriptive quantitative synthesis of effectiveness/predictability and achieved distal movement, stratified by timing of outcome assessment and by distalization protocol.

Stratification Factor	Category	Number of Studies (References	Predictability/Effectiveness Reported	Achieved Distal Movement Reported
Timing of assessment	End of distalization phase	7 [2,5,11,13,15,16,19]	~36.5–88.4% (where reported)	0.84–2.60 mm
Timing of assessment	End of comprehensive treatment	9 [4,6,12,14,17,18,20,21,22]	~35.4–85.1% (where reported)	0.86–4.86 mm
Distalization protocol	V-pattern/sequential distalization	2 [14,16]	36.5–85.1%	0.88–2.98 mm
Distalization protocol	50% sequential distalization	3 [5,11,22]	36.2–79.9%	1.21–1.85 mm
Distalization protocol	Two-thirds sequential distalization	2 [4,20]	Not reported as a percentage	2.25–2.52 mm
Distalization protocol	Unspecified “sequential maxillary distalization”	2 [6,21]	35.4–56.1%	0.84–0.91 mm

**Table 6:** Quality assessment of cohort studies, according to the Newcastle–Ottawa Scale. **** = maximum of 4 stars for Selection of Participants; ** = maximum of 2 stars for Comparability of Groups; *** = maximum of 3 stars for Outcome/Exposure Assessment (Performance Measurement); ★ = one star awarded, indicating that the study met the corresponding quality criterion.

Author/Year	Selection of Participants (****)	Comparability of Groups (**)	Performance Measurement (***)	Overall Score
Simon et al., 2014 [2]	★★★★	★	★★★	8/9
Ravera et al., 2016 [4]	★★★★	★	★★★	7/9
Lin et al., 2023 [6]	★★★★	★	★★★	7/9
Grünheid et al., 2017 [13]	★★★★	★	★★★	8/9
Cui et al., 2022 [14]	★★★★	★	★★★	8/9
Li et al., 2023 [16]	★★★★	★	★★★	8/9
Chen et al., 2023 [18]	★★★★	★	★★★	6/9
Simon et al., 2014 [19]	★★★★	★	★★★	8/9
Caruso et al., 2019 [20]	★★★★	★	★★★	7/9
Mamani et al., 2024 [21]	★★★★	★	★★★	8/9
Liu et al., 2024 [22]	★★★★	★	★★★	8/9

**Table 7:** GRADE Summary-of-Findings table for the certainty of the evidence regarding maxillary molar distalization with clear aligners. GRADE Working Group grades of evidence: ⊕⊕⊕⊕ = High certainty; ⊕⊕⊕◯ = Moderate certainty; ⊕⊕◯◯ = Low certainty; ⊕◯◯◯ = Very low certainty.

Outcome	No. of Studies (Participants)	Study Design	Certainty Rating Considerations (GRADE Domains)	Certainty of Evidence (GRADE)	Summary of Findings
Achieved maxillary molar distalization (mm)	16 studies (461 patients)	Non-randomized studies (11 retrospective cohort; 5 prospective experimental)	Risk of bias: serious (non-randomized designs; moderate confounding per ROBINS-I; inconsistent compliance verification). Inconsistency: serious (achieved movement ranged from 0.84 to 4.86 mm depending on protocol, system, and auxiliaries). Indirectness: serious (different aligner systems, staging protocols, and timing of outcome assessment). Imprecision: serious (small samples; SDs/CIs inconsistently reported). Publication bias: not formally assessed.	⊕◯◯◯ VERY LOW	Clear aligners can produce clinically relevant distal movement of the maxillary molars, but the achieved amount is highly variable and consistently smaller than planned.
Predictability of maxillary molar distalization (%)	16 studies (461 patients)	Non-randomized studies (11 retrospective cohort; 5 prospective experimental)	Risk of bias: serious (as above). Inconsistency: serious (predictability ranged from 31.1% to 88.4%). Indirectness: serious (heterogeneous measurement/superimposition methods and outcome timing). Imprecision: serious (limited samples; wide/absent confidence intervals). Publication bias: not formally assessed.	⊕◯◯◯ VERY LOW	Predictability of maxillary molar distalization with clear aligners is highly variable and cannot be reliably generalized across systems or protocols; a 14-day aligner-change interval may be associated with modestly higher predictability in some studies, but the certainty of this finding is very low.

## Data Availability

Data associated with the study has not been deposited into a publicly available repository. All data will be available upon request. The datasets generated and analyzed during the current study are available upon request.

## Supplementary Materials

The following supporting information can be downloaded at: https://www.mdpi.com/article/10.3390/jcm15145568/s1, Table S1: Excluded studies with reasons, Table S2: PRISMA 2020 Checklist.
