# Supplementary material for: Assessment of the Effectiveness and Predictability of Maxillary Molar Distalization with Clear Aligner Systems: A Systematic Review of the Literature
Source: J Clin Med. 2026 Jul 15;15(14):5568. doi: 10.3390/jcm15145568 (PMC13413332; doi:10.3390/jcm15145568)
Supplement: Supplementary file 1 [file jcm-15-05568-s001.zip › jcm-4408526-supplementary.pdf]

Table S1. Supplementary Table. Excluded studies with specific reasons.

Excluded study	Reason for exclusion
Abu-Arqub S et al., 2023	Systematic review/meta-analysis on the general accuracy of orthodontic tooth movement with clear aligners; not a primary study and does not isolate maxillary molar distalization as a distinct outcome.
Al-Nadawi M et al., 2021	Evaluates the effect of aligner wear protocol on overall tooth-movement efficacy; does not report maxillary molar distalization specifically (cited elsewhere in the manuscript as background discussion reference).
Al-Worafi N et al., 2024	Evaluates general predictability of aligner tooth movement; does not report planned versus achieved maxillary molar distalization.
Auladell A et al., 2022	Evaluates attachments in clear aligner therapy in general; does not isolate maxillary molar distalization outcomes.
Balboni A et al., 2023	Narrative/clinical review on clear aligner therapy; not a primary study reporting molar distalization data.
Bilello G et al., 2022	Evaluates anterior tooth movement (retraction/torque) with clear aligners; no maxillary molar distalization data reported.
Blundell H et al., 2023	Narrative review on clear aligner treatment; not a primary study with quantitative molar distalization outcomes.
Bowman S, 2017	Clinical technique/case-based article on Invisalign treatment planning; no usable quantitative outcome on molar distalization.
Caruso S et al., 2020	Evaluates vertical dimension/occlusal changes with clear aligners in general; does not report planned versus achieved molar distalization.
Charalampakis O et al., 2018	Evaluates general efficacy of aligner therapy in comprehensive treatment; does not isolate maxillary molar distalization.
Dianiskova S et al., 2022	Narrative review on clear aligner biomechanics; not a primary study.
Ercoli F et al., 2014	Case-report/clinical description of aligner treatment; no usable quantitative outcome on molar distalization.
Feltl F and Reistenhofer B, 2017	Descriptive/clinical case article on aligner therapy; does not report distalization in millimeters or percentage predictability.
Feng Y et al., 2020	Evaluates anterior segment retraction with clear aligners; no maxillary molar distalization data.
Greco M et al., 2022	Evaluates general accuracy of tooth movement with clear aligners across multiple tooth types; does not isolate maxillary molar distalization results.
Halitchi L et al., 2020	Narrative review on the use of clear aligners in orthodontics; not a primary study.
Hennessy J and Al-Awadhi E, 2016	Does not answer the PICO question; does not assess maxillary molar distalization.
Izhar A et al., 2019	Does not answer the PICO question; does not assess maxillary molar distalization.
Jia L et al., 2023	Does not answer the PICO question; does not assess maxillary molar distalization.
Karras T et al., 2021	Does not answer the PICO question; only performs mandibular molar distalization.
Kim J et al., 2017	Does not answer the PICO question; does not assess maxillary molar distalization.
Kravitz N et al., 2009	Does not answer the PICO question; only assesses anterior teeth.
Kumar V et al., 2020	Does not answer the PICO question; does not assess maxillary molar distalization.
Kuroda S et al., 2014	Does not answer the PICO question; does not assess maxillary molar distalization.
Levrini L et al., 2022	Does not answer the PICO question; does not assess maxillary molar distalization.
Li H et al., 2021	Distalizes the arch en bloc, not just the molars; no comparison between planned and achieved molar-specific movement.
Lione R et al., 2022	No maxillary molar distalization movements are performed.
Pagani P et al., 2016	Does not answer the PICO question; does not assess maxillary molar distalization.

Excluded study	Reason for exclusion
Ren L et al., 2022	Does not answer the PICO question; does not assess maxillary molar distalization.
Sachdev S et al., 2021	Does not answer the PICO question; only assesses anterior teeth.
Schupp W and Haubrich J, 2016	Does not answer the PICO question; does not assess maxillary molar distalization.
Taffarel I et al., 2022	Only focuses on Class II treatment in general; does not report molar distalization outcomes.
Takara Y et al., 2022	Does not answer the PICO question; does not assess maxillary molar distalization.
Weir T, 2017	Does not answer the PICO question; does not assess maxillary molar distalization.
Wu D et al., 2021	Does not answer the PICO question; only performs mandibular molar distalization.
Zhang Z et al., 2022	Does not answer the PICO question; does not assess maxillary molar distalization.
Zhou G et al., 2023	Does not answer the PICO question; does not assess maxillary molar distalization.
Patterson B et al., 2021	Does not answer the PICO question; does not assess maxillary molar distalization.
Lombardo et al., 2017	Does not report maxillary molar distalization in millimeters or percentage predictability; no comparison between planned and achieved movement.

The excluded studies are recorded along with a specific, study-level reason for exclusion, refined following peer review to move beyond broad categorical statements. In total, 39 full-text records were excluded: 35 for not adequately addressing the PICO question (further specified above as, e.g., no maxillary molar distalization, mandibular distalization only, anterior-teeth-only assessment, en-bloc arch distalization, or narrative/systematic review status) and 4 for failing to properly evaluate molar distalization in a usable quantitative format, consistent with Section 3.1 of the revised manuscript.
